# Breaking the silence: the effectiveness of a dialogue-based intervention on family functioning and psychological distress among young adults in a Chinese context

**DOI:** 10.3389/fpsyg.2026.1937724

**Published:** 2026-09-03

**Authors:** Xiang Li, JingHan Qi, Wai I. Ng, Mingxia Zhu

**Affiliations:** Kiang Wu Nursing College of Macau, Macau, Macao SAR, China

**Keywords:** Chinese families, dialogue-based intervention, life and death education, mental well-being, positive psychology

## Abstract

In many Chinese societies, cultural taboos surrounding death have hindered family communication, leading to psychological distress. This study evaluated a culturally sensitive, dialogue-based workshop aimed at helping adolescents and young adults in Macau enhance their ability to discuss life-and-death topics within the family. By using a quasi-experimental design, 123 participants were assigned to the intervention group (*n* = 59) or the control group (*n* = 64). Baseline and post-intervention data on family functioning (APGAR), psychological distress (PHQ-4), and life attitudes (SF-LAI) were collected. Linear mixed models showed that the intervention group significantly improved in family functioning (*p* = 0.002), significantly reduced psychological distress (*p* = 0.018), while no significant difference was observed in life attitudes. The study results indicate that in a cultural context where such topics are traditionally avoided, family dialogue-based education on life and death can may promote family relationships and mental health.

## 1 Introduction

In many Chinese societies, a deep-rooted cultural tradition of viewing death as a taboo subject often creates significant barriers to open communication within families (Pun et al., 2020). This avoidance is not merely a passive silence but can be an active strategy of “family protectionism,” where family members intentionally withhold information and hide their own feelings to shield loved ones from perceived distress and bad luck (Tian et al., 2026). While this behaviour stems from good intentions, it often leads to unintended negative consequences. When end-of-life issues inevitably arise, this lack of open dialogue can result in misunderstandings, unresolved conflicts, and a family environment where individual needs are secondary to collective expectations (Edmonds et al., 2024; Pun et al., 2023). For younger family members, this experience can be particularly confusing, as they may feel a need to suppress their own grief to protect the family, a phenomenon observed in bereaved Chinese adolescents (Li et al., 2023). This communication breakdown can erode core family functions, such as emotional support, cohesion, and adaptability, which are vital for a healthy family system.

For young adults, who are in a critical life stage of exploring their identity and searching for meaning, the family’s silence on life and death can be particularly challenging. The family is a primary source of values and worldview, and when it fails to provide a space for discussing life’s ultimate questions, it can leave young people feeling lost. Research conducted in Chinese contexts has consistently shown a strong connection between the quality of the parent–child relationship and an adolescent’s development of meaning in life (Shek et al., 2021; Sun et al., 2023). Many Chinese young people place a high value on family well-being as part of their life goals (Wang et al., 2023), the lack of profound communication within a family may lead to the absence of their sense of existence. This intergenerational influence is powerful; for instance, among Asian Americans, the parents’ own sense of meaning in life has been found to be a significant predictor of their children’s (Yu and Chang, 2018). The absence of open dialogue therefore acts as a significant obstacle to young adults forming a mature and positive life attitude.

The combination of a strained family environment and a personal struggle for meaning creates a fertile ground for psychological distress. A growing body of evidence demonstrates a clear link between poor family communication and an increased risk of mental health problems among adolescents. A systematic review by Zapf et al. (2023) confirmed that higher quality parent–child communication, as rated by adolescents themselves, is negatively associated with mental health issues like depression and anxiety. Studies focusing specifically on Chinese adolescents have reinforced this finding, showing that positive family communication serves as a protective factor against psychological distress (Ge et al., 2025; Huang et al., 2023). Conversely, maladaptive and limited emotional communication patterns between parents and adolescents are directly linked to higher levels of depressive symptoms (Zhou et al., 2025). This highlights the critical role that family communication plays in the mental well-being of young people.

Life-and-death education, broadly defined as the study of dying, death, and bereavement (DeSpelder and Strickland, 1996), is an interdisciplinary field rooted in existential and developmental psychology. Existential thought posits that confronting mortality is essential for a meaningful life (Frankl, 1985; Yalom, 1980). For adolescents, who are in a critical stage of identity formation and worldview development (Erikson, 1968), engaging with these concepts can be profoundly beneficial. Through open dialogues about life’s finitude, young people can cultivate a deeper appreciation for life, fostering a more robust sense of purpose. These conversations, held within a supportive family context, provide a crucial opportunity for adolescents to process complex emotions and build a stable self-concept, enhancing their psychological well-being and resilience against future life challenges (Klass, 1996). Despite these benefits, comprehensive life-and-death education for the general adolescent population is notably scarce in China and Macao, with existing programs often targeting specific groups like the elderly or terminally ill. This overlooks the vital developmental need of healthy adolescents to understand these topics. Research confirms that family-based dialogues about mortality significantly bolster adolescent mental health and psychological resilience (Wong and Tomer, 2011). The effectiveness of these conversations is deeply influenced by cultural factors and family beliefs, which shape how adolescents perceive and respond to them. As Kastenbaum and Moreman (2018) suggests, every society possesses a unique “social network space” for confronting death, primarily centered on the family in this context. Therefore, encouraging these discussions within families is a crucial step toward advancing a more holistic societal approach to life-and-death education.

To further explore the intrinsic connection between dialogue on the topic of life and death, and the meaning of life and mental health among teenagers, we drew on two complementary theoretical perspectives: the Family Systems Theory and the Communication Privacy Management (CPM) theory. The Family Systems Theory posits that the family is an emotionally interrelated system, where a change in one member can trigger a “chain reaction” and alter the entire system’s operation (Bowen, 1978). This theory suggests that an individual’s psychological distress may be a manifestation of dysfunctional family patterns. A systematic review also confirms that poor parent–child communication is consistently linked to greater psychological distress in adolescents (Zapf et al., 2023). The family systems theory explains why individual-centered intervention measures can have an impact on the entire family, while the CPM theory elucidates how such changes are initiated. The CPM theory states that sensitive topics such as death are managed through “privacy boundaries” (Petronio, 2002). In many Chinese families, the topic of death is governed by a strong, collectively held privacy boundary, often maintained by cultural taboos and a desire to protect family members (Li et al., 2023; Pun et al., 2020). Our intervention aims to help young people acquire the skills to respect and negotiate, thereby re-adjusting family rules, promoting shared dialogue, and enhancing trust within the relationship. This change in communication patterns is expected to improve the overall function of the family and provide a supportive environment for discussing existential issues, thereby enhancing the sense of meaning among young adults. Ultimately, we believe that alleviating systemic dysfunction and cultivating a sense of meaning contribute to reducing individual psychological distress.

Therefore, this study aims to design and evaluate a culturally-sensitive, dialogue-based workshop for young adults and adolescent’s family members in Macau. The intervention is intended to equip young adults and adolescent’s families with the skills and confidence to initiate conversations about life and death within their families. We hypothesize that, following participation in the workshop, participants will report: (H_1_) a significant improvement in their perceived family function and satisfaction; (H_2_) a more positive and mature personal life attitude; and (H_3_) a significant reduction in symptoms of anxiety and depression.

## 2 Methods

### 2.1 Study design

This study employed a non-randomized, controlled experimental design, also known as a quasi-experimental design. The research protocol was reviewed and approved by the Research Ethics Committee of Kiang Wu Nursing College of Macau (Protocol No. REC-2024.0707). All participants provided informed consent before their inclusion in the study.

### 2.2 Participants

Participants were eligible for inclusion if they identified as either young people or parents of adolescent and young adult. Accordingly, the sample of 123 participants included both young individuals and family members of young individuals.

#### 2.2.1 Inclusion criteria for young adults were

1. Aged between 18 and 25 years.
2. Living with or maintaining frequent communication with family members (e.g., parents, siblings, or other close relatives) to facilitate dialogues on end-of-life topics.
3. Proficient in spoken and written Chinese for communication and record-keeping.

#### 2.2.2 Exclusion criteria for young adults were

1. Extremely strained or distant relationships with family members that would impede effective dialogue on end-of-life issues.
2. Other factors that could interfere with the study process or outcomes, such as excessive academic pressure or scheduling conflicts that prevented participation.

#### 2.2.3 Inclusion criteria for family members were

1. Parents of adolescent or young adult family members aged 14 to 25.
2. Willing and able to actively participate in dialogues on end-of-life topics.
3. Proficient in spoken and written Chinese.

#### 2.2.4 Exclusion criteria for family members were

1. Extremely strained or distant relationships with the participating young adult.
2. Other interfering factors such as significant work-related stress or time constraints that would prevent meaningful participation.

### 2.3 Procedures

Participants were recruited through a combination of online advertisements and on-site posters at the university campus. Group assignment was based on participant preference, which is a key feature of the quasi-experimental design. Young adults who consented to attend the workshop on family end-of-life dialogues were assigned to the intervention group. Those who declined the workshop but agreed to complete the questionnaires at both time points were assigned to the control group.

#### 2.3.1 Intervention group

Participants in the intervention group attended a workshop on family end-of-life dialogues, which consisted of two sessions totaling 6 hours. The workshop incorporated various teaching methods, including lectures, group discussions, and scenario-based role-playing, to encourage reflection on their personal views on life and death and to enhance their communication skills with family members. Following each session, participants were guided and encouraged to initiate a dialogue on these topics with their family members and to complete corresponding dialogue records.

#### 2.3.2 Control group

The control group served as a no-intervention control. Participants in this group did not attend the workshop or receive any specific intervention related to the study’s topic during the research period.

#### 2.3.3 Data collection

Data were collected from both groups at two time points: at baseline before the intervention began (T_0_) and immediately after the intervention period concluded (T_1_). Questionnaires were administered in both digital (online survey) and paper-based formats, depending on participant convenience. To protect participant confidentiality, all collected data were anonymized during the analysis process.

### 2.4 Intervention

The intervention consisted of a two-session, six-hour workshop delivered over two separate days. The workshop was conducted by a single facilitator, a PhD in Psychology with extensive experience in delivering life-and-death education to diverse community groups, particularly adolescents. To ensure intervention fidelity, the same facilitator delivered the identical protocol to all groups. The workshop sessions were designed for included participants, family involvement was facilitated through take-home family dialogue tasks assigned at the end of each session. The detailed structure of the workshop is outlined in the Table 1.

**Table 1:** Structure of the Dialogue-Based Intervention Workshop.

Session	Component	Duration	Content
Session 1: “my” existence and family relationships: initiating key conversations	Lecture & group discussion	2 h	Explored individual life status in relation to family dynamics, common interaction patterns, and the core elements of Nonviolent Communication (NVC).
	Group role-playing & feedback	1 h	Participants practiced NVC skills through scenario-based role-playing simulating common communication challenges within families.
	Family dialogue task	N/A	Participants were instructed to observe and reshape daily communication with family using positive expression and NVC techniques.
Session 2: thinking and expressing “my” life journey and meaning	Lecture & group discussion	1.5 h	Explored multi-faceted interpretations of life and death, emphasizing the significance of discussing these topics within Chinese culture.
	Expressive arts creation	1.5 h	Participants created art (drawing/craft) and a written statement to express their desired state of life and family relationships.
	Family dialogue task	N/A	Participants were encouraged to initiate a conversation with family members about their respective views on life and death by sharing their art piece.

### 2.5 Measures

#### 2.5.1 Life attitude

The Short-form Life Attitude Inventory (SFLAI) was used to assess the life attitudes of the participants (Hsieh and Pan, 2015). This 24-item scale measures six dimensions: Ideals, Autonomy, Love and Care, Sense of Existence, Death Attitude, and Life Experience. The SFLAI has demonstrated good reliability, with a Cronbach’s *α* of 0.93 for the total scale and coefficients ranging from 0.68 to 0.80 for its subscales.

#### 2.5.2 Family function

The Family APGAR Scale is a 5-item screening tool originally developed by Smilkstein (1978) to quickly measure an individual’s satisfaction with their family’s functioning. It assesses five core dimensions: Adaptability, Partnership, Growth, Affection, and Resolve. Each item is rated on a 3-point scale: “almost always” (2 points), “some of the time” (1 point), and “hardly ever” (0 points). The total score ranges from 0 to 10, with scores of 7–10 indicating a highly functional family, 4–6 indicating moderate dysfunction, and 0–3 indicating severe dysfunction.

The scale’s validity and reliability were confirmed in subsequent research (Smilkstein et al., 1982), and it has been widely used across different cultural contexts due to its brevity. Crucially for this study, the Chinese version of the Family APGAR has been validated, demonstrating good reliability and validity in a Chinese-speaking population (Fan, 1999). Its application in a similar research context was shown in a recent study on family caregivers of Chinese adolescents with depression, which confirmed the scale’s adequate internal consistency (Cronbach’s *α* = 0.86) (Zhang et al., 2020).

#### 2.5.3 Psychological distress

The patient health questionnaire-4 (PHQ-4) is an ultra-brief, 4-item screening tool for anxiety and depression developed by Kroenke et al. (2009). It asks participants how often they have been bothered by core symptoms of anxiety (2 items) and depression (2 items) over the past 2 weeks. Each item is scored on a 4-point scale from 0 (“Not at all”) to 3 (“Nearly every day”). The total score ranges from 0 to 12, with higher scores indicating greater psychological distress. The original validation study found the PHQ-4 to have excellent internal consistency (Cronbach’s *α* = 0.85) and strong construct validity (Kroenke et al., 2009). Its reliability and validity have also been established in Chinese populations, confirming it as an effective and efficient screening tool for this study (Zhang et al., 2016).

### 2.6 Data analysis

Data analysis was conducted using R software (version 4.4.2). Descriptive statistics were used to summarize participant characteristics. Normally distributed continuous variables were presented as mean ± standard deviation (SD) and compared using the independent samples t-test. Non-normally distributed continuous variables were presented as median and interquartile range (IQR) and compared using the Mann–Whitney U test. Categorical variables were summarized as frequencies and percentages (*n*, %) and compared using the chi-square test or Fisher’s exact test where appropriate.

To evaluate the effect of the intervention, Linear Mixed Models (LMMs) were employed. These models assessed the change in outcome scores (APGAR, SFLAI, and PHQ-4) from baseline to post-intervention, with terms for time, group (intervention vs. control), and the time-by-group interaction. The interaction term was of primary interest, as it indicates whether the change over time differed significantly between the two groups.

Attrition analyses were conducted by comparing participants who completed follow-up with those lost to follow-up. To address potential selection bias due to the non-randomized design and to account for missing data, Inverse Probability of Weighting (IPW) was applied to the full analysis sample. A logistic regression model was used to estimate each participant’s probability of having complete follow-up data, with data completeness as the dependent variable and baseline covariates (gender, educational attainment, living arrangement, T_0_ APGAR, SFLAI, and PHQ scores) as predictors. The inverse of the estimated probability (1/propensity score) was used as the individual weight in the IPW analyses. To reduce variability, we truncated the extreme weights at the 1st and 99th percentiles. Furthermore, sensitivity analyses were conducted. These included running unweighted models on the full sample and repeating the analysis using only the subset of participants with complete data for both time points.

For all regression models, the regression coefficient (*β*), 95% confidence interval (CI), and *p*-value were reported. A *p*-value of less than 0.05 was considered statistically significant.

## 3 Results

### 3.1 Baseline characteristics

A total of *N* = 123 participants were included in the study, with *n* = *59* in the intervention group and *n* = 64 in the control group. The baseline characteristics of the study participants are displayed in Table 2. At baseline, the APGAR score in the intervention group was lower than that of the control group [5.00 (4.00, 5.50) vs. 7.00 (5.00, 9.25), *p* < 0.001]. Similarly, the SFLAI score was significantly lower in the intervention group than in the control group [131.00 (110.00, 149.00) vs. 149.00 (127.00, 161.50), *p* = 0.002]. A statistically significant difference was observed in educational attainment between the two groups (*p* = 0.017). There were no significant differences between groups for other variables, such as gender, living arrangements, frequency of communication, and history of psychological counseling (*p >* 0.05). In addition, before IPW, the standardized mean differences (SMDs) for most covariates exceeded the conventional threshold of 0.1, with values ranging from 0.032 to 0.826, indicating substantial baseline imbalances between groups. After applying IPW, all SMDs were substantially reduced to below 0.1 (range: <0.001 to 0.110), suggesting that the weighting procedure successfully achieved adequate covariate balance between the two groups.

**Table 2:** Demographic characteristics and baseline measurements of the study participants.

Variables	Total (*N* = 123)	Intervention (*n* = 59)	Control (*n* = 64)	*p* value	SMD before IPW (unadjusted)	SMD after IPW (IPW-adjusted)
Measures						
APGAR_T_0_, median (*Q1*, *Q3*)	5.00 (5.00, 8.00)	5.00 (4.00, 5.50)	7.00 (5.00, 9.25)	<0.001	0.826	0.087
SFLAI_T_0_, Median (*Q1*, *Q3*)	139.00 (119.50, 158.50)	131.00 (110.00, 149.00)	149.00 (127.00, 161.50)	0.002	0.603	0.110
PHQ_T_0_, Median (*Q1*, *Q3*)	4.00 (2.00, 4.50)	4.00 (2.00, 6.00)	4.00 (1.75, 4.00)	0.087	0.332	0.004
Demographics						
Gender (%)				0.112	0.288	0.092
Male	24 (19.51)	15 (25.42)	9 (14.06)			
Female	99 (80.49)	44 (74.58)	55 (85.94)			
Education (%)				0.017	0.603	0.104
High school or below	16 (13.01)	4 (6.78)	12 (18.75)			
College/university (student)	58 (47.15)	36 (61.02)	22 (34.38)			
College/university (graduate)	33 (26.83)	14 (23.73)	19 (29.69)			
Postgraduate or above	16 (13.01)	5 (8.47)	11 (17.19)			
Living together (%)				0.806	0.044	0.005
Yes	95 (77.24)	45 (76.27)	50 (78.12)			
No	28 (22.76)	14 (23.73)	14 (21.88)			
Relationship satisfaction (%)				0.376	0.376	0.059
Very dissatisfied	1 (0.82)	0 (0)	1 (1.56)			
Dissatisfied	3 (2.46)	2 (3.45)	1 (1.56)			
Neutral	24 (19.67)	15 (25.86)	9 (14.06)			
Dissatisfied	56 (45.9)	25 (43.1)	31 (48.44)			
Very satisfied	38 (31.15)	16 (27.59)	22 (34.38)			
Communication frequency (%)				0.375	0.378	0.036
Every day	37 (30.08)	22 (37.29)	15 (23.44)			
4–6 times per week	24 (19.51)	9 (15.25)	15 (23.44)			
1–3 times per week	24 (19.51)	9 (15.25)	15 (23.44)			
Less than once per week	21 (17.07)	11 (18.64)	10 (15.62)			
Less than once per month	17 (13.82)	8 (13.56)	9 (14.06)			
Received psychological counseling (%)				0.861	0.032	0.000
Yes	11 (8.94)	5 (8.47)	6 (9.38)			
No	112 (91.06)	54 (91.53)	58 (90.62)			
Marital status (parent) (%)				0.178	0.513	0.085
Unmarried	1 (2.13)	0 (0)	1 (3.45)			
Married and living with spouse	40 (85.11)	14 (77.78)	26 (89.66)			
Separated/divorced/Widowed	6 (12.77)	4 (22.22)	2 (6.9)			
Kinship (parent) (%)				0.073	0.640	0.066
Father	3 (6.38)	3 (16.67)	0 (0)			
Mother	39 (82.98)	13 (72.22)	26 (89.66)			
Other	5 (10.64)	2 (11.11)	3 (10.34)			

Continuous variables are presented as median (interquartile range, Q1–Q3) unless otherwise indicated; categorical variables are presented as n (%). *N* = total sample size; *n* = subsample size.

### 3.2 Attrition analysis

Of the 123 participants enrolled at baseline, 35 completed the follow-up assessment and 88 were lost to follow-up (attrition rate: 71.54%). The follow-up completion rate was significantly higher in the intervention group than in the control group (44.07% vs. 14.06%, *p* < 0.001). Comparisons between completers and non-completers showed no significant differences in baseline APGAR, SFLAI, PHQ-4 scores, or most demographic characteristics (all *p* > 0.05), except for communication frequency (*p* = 0.015). These findings suggest that although differential attrition existed between groups, baseline outcome measures were generally comparable. IPW was therefore applied to account for potential attrition bias (Supplementary Tables S1, S2).

### 3.3 Effect of intervention on outcomes

We employed linear mixed models (LMMs) with inverse probability weighting (IPW) to analyze the intervention effects on SFLAI, APGAR, and PHQ scores.

#### 3.3.1 Intervention effects on SFLAI scores

The results for the Short-form Life Attitude Inventory (SFLAI) are presented in Table 3. Linear mixed models (LMMs) indicated no significant difference in SFLAI scores between the intervention and control groups, after adjusting for gender, educational attainment, communication frequency, history of psychological counseling, and baseline APGAR, SFLAI, and PHQ scores (*p* = *0.938*). Furthermore, neither the main effect of time nor the group-by-time interaction effect reached statistical significance (*p* = 0.874 and *p* = 0.097, respectively).

**Table 3:** Linear mixed model analysis of SFLAI scores (*N* = 123).

Variables	*β* (95% CI)	*p*-value	Std*.β*
Group			
Control group	Ref.		Ref.
Intervention group	0.077 (−1.864 to 2.018)	0.938	0.001
Time			
T0	Ref.		Ref.
T1	0.315 (−3.569 to 4.199)	0.874	0.005
Group*time (Intervention×T1)	3.833 (−0.659 to 8.324)	0.097	0.055
Gender			
Male	Ref.		Ref.
Female	0.267 (−1.661 to 2.195)	0.786	0.004
Education level			
High school or below	Ref.		Ref.
College/university (student)	−0.652 (−2.660 to 1.357)	0.526	−0.013
College/university (graduate)	−0.768 (−3.002 to 1.466)	0.501	−0.013
Postgraduate or above	−2.758 (−5.894 to 0.377)	0.087	−0.038
Received psychological counseling (%)			
Yes	Ref.		Ref.
No	1.569 (−1.146 to 4.284)	0.259	0.017
Communication frequency (%)			
Every day	Ref.		Ref.
4–6 times per week	0.492 (−1.692 to 2.676)	0.659	0.007
1–3 times per week	0.679 (−1.509 to 2.867)	0.544	0.011
Less than once per week	−0.058 (−2.686 to 2.569)	0.965	−0.001
Less than once per month	−0.266 (−2.807 to 2.274)	0.837	−0.003
APGAR_T0	0.029 (−0.335 to 0.393)	0.874	0.003
SFLAI_T0	0.969 (0.929 to 1.010)	<0.001	0.968
PHQ_T0	−0.243 (−0.604 to 0.118)	0.190	−0.025

Values are unstandardized regression coefficients (*β*) with 95% confidence intervals (CI) from linear mixed models. Std.*β* = standardized regression coefficient; Ref. = reference group. *n* = 123 (total sample size).

#### 3.3.2 Intervention effects on APGAR scores

The results for the APGAR are presented in Table 4. After adjusting for relevant covariates, a significant group-by-time interaction effect was observed (*p* = 0.002), indicating that the trajectory of APGAR scores over time differed between the intervention and control groups. Furthermore, participants with no history of psychological counseling exhibited significantly higher APGAR scores compared with those who had received counseling (*β* = 0.804, *p* = 0.031).

**Table 4:** Linear mixed model analysis of APGAR scores (*N* = 123).

Variables	*β* (95% CI)	*p*-value	Std.*β*
Group			
Control	Ref.		Ref.
Intervention	−0.246 (−0.762 to 0.270)	0.351	−0.045
Time			
T_0_	Ref.		Ref.
T_1_	−0.924 (−1.956 to 0.109)	0.082	−0.140
Group*time (intervention×T_1_)	1.949 (0.755 to 3.143)	0.002	0.264
Gender			
Male	Ref.		Ref.
Female	0.142 (−0.371 to 0.654)	0.588	0.020
Education			
High school or below	Ref.		Ref.
College/university (student)	0.006 (−0.528 to 0.540)	0.982	0.001
College/university (graduate)	0.261 (−0.333 to 0.854)	0.391	0.042
Postgraduate or above	0.292 (−0.541 to 1.126)	0.493	0.038
Psychological counseling			
Yes	Ref.		Ref.
No	0.804 (0.082 to 1.525)	0.031	0.081
Communication frequency			
Every day	Ref.		Ref.
4–6 times per week	0.079 (−0.501 to 0.659)	0.790	0.011
1–3 times per week	0.038 (−0.544 to 0.619)	0.899	0.006
Less than once per week	−0.427 (−1.125 to 0.271)	0.233	−0.056
Less than once per month	−0.044 (−0.719 to 0.632)	0.899	−0.005
APGAR_T0	0.866 (0.769 to 0.963)	< 0.001	0.851
SFLAI_T0	0.001 (−0.010 to 0.012)	0.834	0.011
PHQ_T0	0.026 (−0.070 to 0.122)	0.600	0.025

Values are unstandardized regression coefficients (*β*) with 95% confidence intervals (CI) from linear mixed models. Std.β = standardized regression coefficient; Ref. = reference group. *n* = 123 (total sample size).

#### 3.3.3 Intervention effects on PHQ scores

The results for the Patient Health Questionnaire (PHQ) are presented in Table 5. After adjusting for relevant covariates, a significant group-by-time interaction effect was observed (*p* = 0.018), suggesting that the trajectory of PHQ scores over time differed significantly between the intervention and control groups.

**Table 5:** Linear mixed model analysis of PHQ scores (N = 123).

Variables	*β* (95% CI)	*p*-value	Std*.β*
Group			
Control	Ref.		Ref.
Intervention	0.106 (−0.237 to 0.450)	0.546	0.020
Time			
T0	Ref.		Ref
T1	0.640 (−0.048 to 1.327)	0.070	0.103
Group*time (intervention×T1)	−0.972 (−1.767 to −0.178)	0.018	−0.140
Gender			
Male	Ref.		Ref
Female	0.269 (−0.072 to 0.610)	0.125	0.041
Education			
High school or below	Ref.		Ref
College/university (student)	0.164 (−0.192 to 0.519)	0.368	0.032
College/university (graduate)	−0.001 (−0.396 to 0.395)	0.998	−0.000
Postgraduate or above	0.040 (−0.515 to 0.595)	0.887	0.006
Psychological counseling			
Yes	Ref.		Ref
No	−0.252 (−0.733 to 0.228)	0.305	−0.027
Communication frequency			
Every day	Ref.		Ref
4–6 times per week	0.080 (−0.306 to 0.467)	0.684	0.012
1–3 times per week	0.162 (−0.225 to 0.549)	0.414	0.025
Less than once per week	0.244 (−0.221 to 0.709)	0.305	0.034
Less than once per month	0.101 (−0.348 to 0.551)	0.660	0.012
APGAR_T0	0.021 (−0.043 to 0.085)	0.525	0.022
SFLAI_T0	−0.002 (0.009 to 0.005)	0.624	−0.018
PHQ_T0	0.922 (0.858 to 0.985)	<0.001	0.938

Values are unstandardized regression coefficients (β) with 95% confidence intervals (CI) from linear mixed models. Std.β = standardized regression coefficient; Ref. = reference group. *n* = 123 (total sample size).

#### 3.3.4 Robustness analysis

To evaluate the robustness of our findings, we conducted sensitivity analyses by re-fitting the linear mixed models (LMMs) based on two alternative datasets: the unweighted total sample (*N* = 123) and the matched complete-case sample (*n* = 35). The results are presented in Table 6.

**Table 6:** Sensitivity analysis of intervention effects.

Outcome	Method	Variables	*β* (95% CI)	*p*-value
SF-LAI	Unweighted			
		Group (intervention vs. control)	−0.283 (−2.352 to 1.786)	0.789
		Time (T1 vs. T0)	−2.831 (−6.550 to 0.888)	0.138
		Group*time (intervention × T1)	6.948 (2.542 to 11.354)	0.002
	Complete-case			
		Group (intervention vs. control)	−1.856 (−9.348 to 5.637)	0.630
		Time (T1 vs. T0)	−3.222 (−10.295 to 3.851)	0.378
		Group*time (intervention × T1)	7.299 (−0.907 to 15.505)	0.091
APGAR	Unweighted			
		Group (intervention vs. control)	−0.247 (−0.784 to 0.291)	0.370
		Time (T1 vs. T0)	−0.439 (−1.404 to 0.527)	0.374
		Group*time (intervention × T1)	1.707 (0.563 to 2.851)	0.004
	Complete-case			
		Group (intervention vs. control)	−0.899 (−2.655 to 0.858)	0.320
		Time (T1 vs. T0)	−0.333 (−2.096 to 1.430)	0.712
		Group*time (intervention × T1)	1.641 (−0.405 to 3.687)	0.122
PHQ	Unweighted			
		Group (intervention vs. control)	0.082 (−0.273 to 0.436)	0.653
		Time (T1 vs. T0)	0.698 (0.060 to 1.335)	0.034
		Group*time (intervention × T1)	−0.937 (−1.693 to −0.182)	0.016
	Complete-case			
		Group (intervention vs. control)	0.520 (−0.736 to 1.776)	0.422
		Time (T1 vs. T0)	0.778 (−0.461 to 2.017)	0.227
		Group*time (intervention × T1)	−1.047 (−2.484 to 0.390)	0.163

Values are unstandardized regression coefficients (β) with 95% confidence intervals (CI) from linear mixed models. Unweighted = sensitivity analysis using the full sample without weighting; Complete case = sensitivity analysis restricted to participants with complete data at both time points.

Regarding the SFLAI, the unweighted analysis revealed a significant group-by-time interaction effect (*p* = 0.002), suggesting a significant difference in the trajectory of SFLAI scores over time between the two groups. In the matched complete-case sample, neither the main effect of group (*p* = 0.630) nor the interaction effect (*p* = 0.091) reached statistical significance, likely due to the reduced sample size, however, the point estimate of the interaction remained consistent with the unweighted results.

For the APGAR, the unweighted analysis yielded a significant group-by-time interaction effect (*p* = 0.004), which is consistent with our primary IPW-weighted findings. While the interaction effect did not reach significance in the matched complete-case sample (*p* = 0.122), the direction of the effect remained consistent.

For the PHQ, the unweighted analysis showed a significant group-by-time interaction effect (*p* = 0.016), consistent with the IPW-weighted results. Although the interaction effect was not statistically significant in the matched complete-case sample (*p* = 0.163), the direction of the effect was consistent across all three analytical approaches (IPW-weighted, unweighted, and complete-case).

Overall, the IPW-weighted results were largely consistent with the unweighted results. Some effects in the complete-case analyses did not reach statistical significance due to the reduced sample size, but the directions of the effects remained consistent, suggesting that the findings were generally consistent across different analytical approaches. However, these results should be interpreted cautiously given the substantial loss to follow-up and reduced sample size in the complete-case analysis. Although the unweighted analysis showed a statistically significant group-by-time interaction for SFLAI, this effect was attenuated after applying IPW adjustment and did not reach statistical significance in the primary analysis. This discrepancy suggests that baseline imbalance and potential selection bias may have influenced the unweighted estimation.

## 4 Discussion

This study aimed to evaluate the effectiveness of a culturally sensitive, dialogue-based workshop designed to empower young adults in Macau to initiate conversations about life and death within their families. The findings provide preliminary evidence that the intervention was associated with significant improvements in perceived family functioning and reductions in psychological distress, although its impact on life attitude was not statistically significant in the primary analysis. This discussion will interpret these findings, address the study’s methodological nuances and limitations, and propose directions for future research.

### 4.1 Principal findings and intervention efficacy

The primary finding of this study is that the workshop successfully improved participants’ perceived family functioning (APGAR) and reduced their symptoms of anxiety and depression (PHQ-4), thus supporting hypotheses H_1_ and H_3_. The significant group-by-time interaction effect for both APGAR (*p* = 0.002) and PHQ-4 scores (*p* = 0.018) indicates that the intervention group in the IPW-weighted analysis indicates that the intervention group experienced positive changes that were not observed in the control group. This outcome is consistent with theoretical frameworks linking open family communication to enhanced mental well-being (Zapf et al., 2023). By equipping young adults with skills to broach the culturally taboo topic of death (Chen et al., 2024; Xie et al., 2024), the workshop likely served as a catalyst for more supportive family interactions, thereby strengthening family function.

### 4.2 Interpreting the null finding for life attitude

In contrast, our second hypothesis (H_2_) that the workshop would foster a more positive life attitude (SFLAI), which was not supported in the primary IPW-weighted analysis (*p* = 0.097). This result, while borderline, may reflect that life attitude is a relatively robust personal characteristic (Valdés-Stauber et al., 2021). A brief, six-hour workshop may be sufficient to impart communication skills that yield immediate relational benefits, but insufficient to fundamentally alter one’s philosophical outlook. This interpretation is supported by evidence that while meaning-centered interventions can improve a sense of meaning, the durability of effects is often inconsistent and under-studied in the short term (Shen et al., 2025; Tian et al., 2024). For instance, one randomized trial that successfully increased life attitude scores in cancer patients used a more intensive eight-week positive psychotherapy program (Saeedi et al., 2019).

It should be noted that the sensitivity analyses revealed some inconsistency regarding the SFLAI outcome. Specifically, the unweighted analysis demonstrated a statistically significant group-by-time interaction (*p* = 0.002), whereas the IPW-weighted primary analysis did not reach statistical significance (*p* = 0.097). This difference may be attributable to the baseline imbalance between groups, as participants in the intervention group had significantly lower baseline SFLAI scores compared with controls. Because participants self-selected into the intervention condition, the unweighted model may have partially reflected pre-existing differences between groups rather than intervention effects alone. The IPW approach was therefore adopted as the primary analysis to reduce potential selection bias by balancing observed baseline characteristics. Importantly, although statistical significance varied across analytical approaches, the estimated direction of the intervention effect remained positive and consistent, with the complete-case analysis showing a similar effect estimate. Therefore, these findings should be interpreted cautiously as preliminary evidence suggesting potential improvement in life attitudes, rather than definitive evidence of intervention efficacy on SFLAI.

### 4.3 Interpreting the mechanisms of change

A novel contribution of this study is its exploration of a “bottom-up” approach to fostering family change. The literature notes that youth-initiated change is an underexamined area, with most research focusing on parent-to-child influence (Vaccaro et al., 2021). This model empowers young adults to act as initiators of difficult conversations, positioning them as potential agents of change within the family system. This youth-centered dynamic is an area that remains relatively underexamined, as much of the literature has traditionally focused on parent-to-child influence. Our approach aligns with models in other fields that aim to build family competence from the everyday context rather than imposing a top-down expert model (Fisher et al., 2020), suggesting a promising pathway for navigating sensitive cultural topics.

While our results are promising, the precise mechanisms driving these changes were not measured in this study. Our theoretical model posits that the workshop imparted communication skills that facilitated actual family dialogues, which in turn improved relational quality and reduced distress. However, this study operated as a “black box” evaluation; we measured the inputs (the workshop) and the outputs (APGAR and PHQ-4 scores), but not the intermediate processes such as changes in communication quality or the content of the dialogues themselves. This gap between demonstrating an intervention’s efficacy and understanding its mechanisms of change remains a central challenge in psychotherapy research (Lemmens et al., 2022). Therefore, we can only infer, not confirm, this causal pathway.

Furthermore, the pattern of findings suggests the intervention’s benefits may be more relational than existential. The significant improvement in family functioning (H_1_) and the concurrent reduction in psychological distress (H_3_), contrasted with the non-significant change in life attitude (H_2_), implies that the primary active ingredient of the intervention may have been the reduction of interpersonal friction and the bolstering of perceived family support. This interpretation aligns with extensive research showing that a supportive family environment acts as a direct protective factor against psychological distress (Ge et al., 2025; Huang et al., 2023).

### 4.4 Strengths and limitations

This study has several strengths. Its culturally-sensitive design is paramount, as evidence shows that culturally adapted interventions are more effective for Chinese and other Asian families (Ali et al., 2023; Xu et al., 2025). The use of a control group and advanced statistical methods like IPW also enhances the findings’ credibility.

However, the study’s limitations must be acknowledged. The most significant is the quasi-experimental design, which introduces a risk of selection bias. Although IPW was used to reduce observed baseline differences, residual confounding from unmeasured characteristics (e.g., motivation toward life-death education) cannot be completely excluded. A future Randomized Controlled Trial (RCT) would be necessary to establish a more definitive causal link. Second, the sample of young adults in Macau may limit generalizability. Third, the reliance on self-report measures may be subject to social desirability bias. Finally, the short-term follow-up is a critical limitation. Meta-analyses of similar interventions show that while initial benefits are common, they may not persist at 2 to 6 month follow-ups, and long-term data remains a major gap in the literature (Shen et al., 2025; Kim et al., 2024). Our study cannot assess the long-term sustainability of the observed effects. The substantial loss to follow-up may have introduced potential attrition bias. Although baseline outcome measures were comparable between completers and non-completers, differential retention between groups cannot be completely excluded. Future studies should adopt strategies to improve follow-up completion.

### 4.5 Implications and future research

Despite these limitations, our findings have important implications. Practically, this workshop presents a scalable model for preventive mental health care that can be implemented in university and community settings, which are increasingly becoming sites for death education in China (Wang et al., 2023; Xu et al., 2022). It offers a concrete tool to address the pervasive cultural silence around end-of-life issues, a known source of family distress (Tian et al., 2026; Li et al., 2023).

For future research, RCT is the next logical step. Longitudinal follow-up is crucial to evaluate the durability of effects, particularly given the evidence of waning benefits in other meaning-centered interventions (Shen et al., 2025). Future studies must also measure potential mediating variables directly, such as changes in family communication quality and frequency and empirically test the proposed theoretical pathway (Lemmens et al., 2022). Efforts should also be made to recruit a more gender-balanced sample to enhance generalizability. Additionally, researchers should also consider testing interventions where the family itself is the main unit of analysis, a design that remains scarce in the literature on Chinese family communication (Ali et al., 2023). Finally, incorporating a qualitative component would provide rich, nuanced data on the process of change and the specific ways in which family dynamics were altered.

## 5 Conclusion

In conclusion, this study provides preliminary evidence that a culturally sensitive, dialogue-based workshop can be an effective intervention for young adults in a Chinese context. By empowering them with the skills to navigate difficult but essential conversations, the findings provide preliminary evidence that a culturally sensitive dialogue-based workshop may be associated with improved family functioning and reduced psychological distress. This research addresses a notable gap by demonstrating the viability of a youth-centered, “bottom-up” approach to fostering healthier family dynamics. It underscores the critical role of open communication in family well-being and offers a promising avenue for promoting mental health within a cultural framework where such topics are often avoided.

## Data Availability

The original contributions presented in the study are included in the article/supplementary material, further inquiries can be directed to the corresponding author.
