# Supplementary material for: Breaking the silence: the effectiveness of a dialogue-based intervention on family functioning and psychological distress among young adults in a Chinese context
Source: Front Psychol. 2026 Sep 3;17:1937724. doi: 10.3389/fpsyg.2026.1937724 (PMC13581623; doi:10.3389/fpsyg.2026.1937724)
Supplement: Supplementary file 1 [file Supplementary_file_1.pdf]

Table S1. Attrition proportion between groups

Variables	Total (*N = 123*)	Intervention (*n = 59*)	Control (*n = 64*)	*P* value
Follow-up Completion (%)				< 0.001
**Dropout Group**	88 (71.54)	33 (55.93)	55 (85.94)	
Completion group	35 (28.46)	26 (44.07)	9 (14.06)	

Table S2. Comparison of baseline characteristics between completers and dropouts

Variables	Total (*N = 123*)	**Dropout Group** (n= 88)	Completion group (*n* = 35)	*P* value
APGAR_T0, Median (Q1,Q3)	5.00 (5.00, 8.00)	6.00 (5.00, 9.00)	5.00 (5.00, 7.50)	0.440
SFLAI_T0, Median (Q1,Q3)	139.00 (119.50, 158.50)	139.50 (119.25, 160.00)	138.00 (120.00, 156.50)	0.799
PHQ_T0, Median (Q1,Q3)	4.00 (2.00, 4.50)	4.00 (2.00, 4.00)	3.00 (2.00, 5.00)	0.390
**Gender (%)**				0.676
Male	24 (19.51)	18 (20.45)	6 (17.14)	
Female	99 (80.49)	70 (79.55)	29 (82.86)	
**Education (%)**				0.117
High school or below	16 (13.01)	14 (15.91)	2 (5.71)	
College/university (student)	58 (47.15)	43 (48.86)	15 (42.86)	
College/university (graduate)	33 (26.83)	23 (26.14)	10 (28.57)	
Postgraduate or above	16 (13.01)	8 (9.09)	8 (22.86)	
**Living together (%)**				0.059
Yes	95 (77.24)	64 (72.73)	31 (88.57)	
No	28 (22.76)	24 (27.27)	4 (11.43)	
**Relationship satisfaction (%)**				0.955
Very dissatisfied	1 (0.82)	1 (1.14)	0 (0)	
Dissatisfied	3 (2.46)	2 (2.27)	1 (2.94)	
Neutral	24 (19.67)	18 (20.45)	6 (17.65)	
Dissatisfied	56 (45.9)	41 (46.59)	15 (44.12)	
Very dissatisfied	38 (31.15)	26 (29.55)	12 (35.29)	
**Communication frequency (%)**				0.015
Every day	37 (30.08)	20 (22.73)	17 (48.57)	
4–6 times per week	24 (19.51)	18 (20.45)	6 (17.14)	
1–3 times per week	24 (19.51)	16 (18.18)	8 (22.86)	
Less than once per week	21 (17.07)	18 (20.45)	3 (8.57)	
Less than once per month	17 (13.82)	16 (18.18)	1 (2.86)	
**Received psychological counseling (%)**				0.659
Yes	11 (8.94)	9 (10.23)	2 (5.71)	
No	112 (91.06)	79 (89.77)	33 (94.29)	
**Marital status (parent) (%)**				0.097
Unmarried	1 (2.13)	1 (3.23)	0 (0)	
Married and living with spouse	40 (85.11)	24 (77.42)	16 (100)	
Separated/Divorced/Widowed	6 (12.77)	6 (19.35)	0 (0)	
**Kinship (parent) (%)**				0.232
Father	3 (6.38)	2 (6.45)	1 (6.25)	
Mother	39 (82.98)	24 (77.42)	15 (93.75)	
Other	5 (10.64)	5 (16.13)	0 (0)	
